# Safety Assessment of Total Phenols From Deoiled *Cinnamomum Longepaniculatum* Leaves

**DOI:** 10.1002/fsn3.70008

**Published:** 2025-05-13

**Authors:** Shuting Li, Xi Zhou, Yu Jiang, Manling Fu, Yefei Yuan, Xianhong Ou

**Affiliations:** ^1^ Department of Pharmaceutical Sciences School of Pharmacy, Southwest Medical University Luzhou Sichuan China; ^2^ Science and Technology Department Southwest Medical University Luzhou Sichuan China; ^3^ Key Laboratory of Medical Electrophysiology of the Ministry of Education, Medical Electrophysiological Key Laboratory of Sichuan Province Institute of Cardiovascular Research, Department of Cardiology of the Affiliated Hospital, Southwest Medical University Luzhou Sichuan China

**Keywords:** 28‐day oral toxicity study, acute toxicity study, three genetic toxicity tests, total phenols from the deoiled *Cinnamomum longepaniculatum* leaves

## Abstract

Total phenols from the deoiled *Cinnamomum longepaniculatum* leaves (DCL‐TP) is a phenolic compound that exhibits a variety of strong biological activities, especially antioxidant activity due to their phenolic hydroxyl structure characteristics, so DCL‐TP is attaining improved relevance and acceptance. However, a lack of toxicological information limits its application. The present study aimed to assess the toxicological profile of DCL‐TP, through an acute toxicity study, 28‐day oral toxicity study, and three genetic toxicity tests, which laid a foundation for the development of DCL‐TP as an antilipid oxidant and provided a basis for pharmacological application. In the acute toxicity study, 10 female and 10 male KM mice were administered by oral gavage 10 g/kg body weight (bwt) of DCL‐TP for 14 days. In the bacterial reverse mutation test (Ames test), the mutagenicity of DCL‐TP was investigated by the plate infiltration method with the number of bacterial reverse mutations as the observation index. Mammal erythrocytes micronucleus test in 25 female and 25 male KM mice and spermatocyte chromosomal aberrations test in 25 male KM mice were randomly assigned to five groups (daily oral dose of 5, 2.5 and 1.25 g/kg bwt). In the 28‐day oral toxicity study, 20 female and 20 male SD rats were randomly assigned to four groups (daily oral dose of 2.5, 1.25 and 0.625 g/kg bwt). No death occurred, poisoning and no adverse effects were observed, indicating the LD50 was higher than 10 g/kg bwt. The Ames test suggested that DCL‐TP had no mutagenicity. There was no significant difference in the number of mammal erythrocytes micronucleus and spermatocyte chromosomes between DCL‐TP and the negative control group (*p* > 0.05). In the 28‐day oral toxicity study, no significant damage or organ abnormalities were observed compared to negative control. Food consumption, body weight, organ weight, urinalysis, blood routine index, blood biochemical index, and histopathology showed normal histology comparable to the control group (*p* > 0.05). This study revealed that DCL‐TP showed no significant toxic effects and no mutagenicity potential genotoxicity. Further chronic toxicological evaluation would be needed to determine its safety and application value.

## Graphical abstract

The present study aimed to assess the toxicological profile of DCL‐TP, through an acute toxicity study, a 28‐day oral toxicity study, and three genetic toxicity tests, which laid a foundation for the development of DCL‐TP as an antilipid oxidant and provided a basis for pharmacological application.

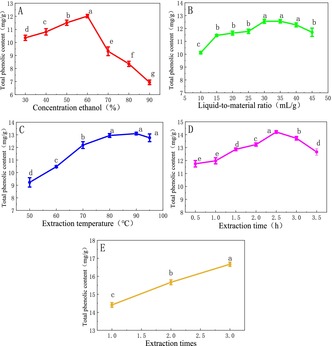

## 1 Introduction

In recent years, food safety has been directly related to physical health and life safety. With the increasing demand for health, the safety of food additives, especially antilipid oxidants, has attracted more and more attention. Moreover, safety concerns such as teratogenic and carcinogenic with synthetic antilipid oxidants augmented the interest in natural antilipid oxidants. The current health care appears to be shifting to traditional medicine with safety (Kahl and Kappus 1993). In the previous research of the research group, the in vitro antioxidant and antilipid oxidation function of DCL‐TP (Total phenols from the deoiled *Cinnamomum longepaniculatum* leaves) were studied, which showed strong potential in antilipid oxidation of food additives.

*C. longepaniculatum* is an important woody incense plant that has been extensively cultivated in Yibin, Sichuan Province, China. It is distributed in Jiangxi, Guangxi, Guangdong, Hunan, Yunnan, and other places (Lin 2020; Zhou and Lian 2020). The substances isolated from this plant include proanthocyanidins (Hu et al. 2019), essential oils (Yuan 2021), polysaccharides (Du et al. 2015), flavonoids (Du et al. 2016), coumarins, glycosides, organic acids (Hu, Luo, and Dai 2019), proteins, and other chemical components (Du et al. 2014). Modern pharmacological studies have shown that these chemical components in deoiled *C. longepaniculatum* leaves have various physiological activities such as antibacterial (Feng et al. 2023), anti‐inflammatory (Tao 2011), analgesic (Chao et al. 2013), anticancer (Zhou et al. 2022), and antioxidation (Chen et al. 2021). At present, the research on deoiled *C. longepaniculatum* leaves at home and abroad mainly focuses on the essential oil, the extraction process of essential oil, and its chemical composition analysis (Ying et al. 2020). Total phenol is one of the important active ingredients in deoiled *C. longepaniculatum* leaves. Therefore, deoiled *C. longepaniculatum* leaves can be used as the main source of phenols. It is cheap and rich in raw materials, and has important research and development values. However, there are few studies on the effective components of nonessential oils such as total phenols of deoiled *C. longepaniculatum* leaves and their safety evaluation, and there is still a lack of reports on the toxicological evaluation of the edible safety of DCL‐TP as an antilipid oxidant.

Therefore, in this study, preliminary studies on the safety of DCL‐TP, and the toxicology experiment are carried out according to the National Food Safety Standard food safety toxicology evaluation procedure (National Standardization Administration Committee, China, GB 15193.1–2014) (National Food Safety Standard Food Safety Toxicology Evaluation Procedure 2014). The acute oral toxicity test of mice, three genetic toxicity tests (bacterial reverse mutation test, mammalian erythrocyte micronucleus test, and mouse spermatocyte chromosome aberration test), and 28‐day oral toxicity test of rats were gavaged with DCL‐TP suspension to KM mice and SD rats, which provide the experimental basis for DCL‐TP as an antilipid oxidation food additive.

## 2 Materials and Methods

### 2.1 Materials

Cyclophosphamide (F131S206786), carboxymethyl cellulose (C14322016), dimethyl sulfoxide (DMSO) (1121E0323), calf serum (220308), Giemsa staining solution (Giemsa) (2306001), and Eosin staining solution (YE2080) were brought from the Shanghai Yuanye Biotechnology Co. Ltd., Shanghai McLean Biochemical Technology Co. Ltd., Beijing Soleibao Technology Co. Ltd., Guangzhou Hongquan Biotechnology Co. Ltd., Beijing Soleibao Technology Co. Ltd., and Bomei Biotechnology Company, respectively. Biochemical incubator (SPX‐150), Low‐speed refrigerated centrifuge (KDC‐2046), Rotary slicer (Leica‐2016), and Tissue embedding machine (BMJ‐A) were purchased from the Shanghai Haixiang Instrument and Equipment Factory, Anhui Zhongke Zhongjia Scientific Instrument Co. Ltd., Leica, Germany, and Changzhou Suburb Zhongwei Electronic Instrument Factory.

### 2.2 Plant Materials

The deoiled *C. longepaniculatum* leaves were brought from the Yibin (Sichuan, China), which were authenticated by Prof. Pixian Shui, the School of Pharmacy, Southwest Medical University (Voucher specimen number SMU/220215–1).

### 2.3 Extraction, Purification, and Identification of DCL‐TP

In extraction procedure, a single‐factor experiment combined with the orthogonal experiment method (Zhou, Jiang, and Yuan 2024) was used to investigate the process parameters of DCL‐TP extraction by heating reflux extraction. According to Table 1, the single‐factor experiment was carried out by the heating reflux extraction method. Then, on the basis of the single‐factor experiment, the extraction of DCL‐TP was carried out according to the L_18_(3^7^) orthogonal experiment, and the factor levels are shown in Table 2.

**TABLE 1:** Single‐factor experimental design of heating reflux extraction.

Factor	Level	Set condition
A concentration ethanol (%)	30. 40. 50. 60. 70. 80. 90	20 mL/g 80°C. 1.5 h. 1 time
B liquid‐to‐material ratio (mL/g)	10. 15. 20. 25. 30. 35. 40. 45	60%. 80°C. 1.5 h. 1 time
C extraction temperature (°C)	50. 60. 70. 80. 90. 95	60%. 35 mL/g. 1.5 h. 1time
D extraction time (h)	0.5. 1. 1.5. 2. 2.5. 3	60%. 35 mL/g. 90°C. 1time
E extraction times	1. 2. 3	60%. 35 mL/g. 90°C. 2.5 h

**TABLE 2:** Factor‐level table of orthogonal experiment.

Level	A (%)	B (mL/g)	C (°C)	D (h)	E (times)
	Concentration ethanol	Liquid‐to‐material ratio	Temperature	Time	Times
1	50	30	80	2	1
2	60	35	90	2.5	2
3	70	40	95	3	3

The isolation of DCL‐TP was carried out by the purification process of phenolic acids in the roots of *Salvia deserta* in Xinjiang (Ren et al. 2021). HPD‐600 resin was selected for the subsequent purification and separation of DCL‐TP. The adsorption kinetics of DCL‐TP by HPD‐600 resin and the static parameters (pH of sample solution; sample concentration) and dynamic parameters (sample flow rate; elution flow rate; elution pH; eluant concentration; elution volume) were investigated.

For the identification of plant phenols, among the commonly used methods, the Folin‐Ciocalteu method (Wang et al. 2016) is used for the determination of total phenol content. The Folin‐Ciocalteu colorimetric method is the most commonly used method for the determination of total phenol content in the Folin‐Ciocalteu method. In the Folin‐Ciocalteu colorimetric method, most of the standard curves were drawn with gallic acid as the standard substance, and the total phenol content was expressed as gallic acid equivalent. When HPD‐600 was used to separate and purify DCL‐TP, the ninhydrin reaction was negative, indicating that the impurities of amino acids and polypeptides had been removed. The Molish reaction of the eluent was negative and clarified, indicating that the polysaccharide impurities had been basically removed. The method of Folin‐Ciocalteus was used to determine the content of DCL‐TP 0.5 mL gallic acid reference solution was taken in a 10‐mL volumetric flask, and 0.6 mL of Folin‐Ciocalteu test solution was added. After 3 min, 1.2 mL of 10% sodium carbonate solution was added, fully shaken, constant volume, 30°C water bath for 1 h, and the corresponding solvent was used as blank. Scanning was performed in the range of 500–900 nm, and the UV scanning of the DCL‐TP extract was performed in the same way. The standard curve was made to determine the total phenol extraction amount and the precision test, repeatability test, stability test, and sample recovery test were carried out.

### 2.4 Animals

The Southwest Medical University's Ethics Review Committee (SMUERC, ethics approval number: 20221005–005) granted the ethics clearance approval for this study. Specific pathogen‐free (SPF) KM mice at average body weights of 18–22 g and 50–100 g for SD rats, purchased from the Experimental Animal Center of Southwest Medical University (Sichuan China license No. SYXK 2023–0017; animal experimental facility license No. SYXK 2023–0065), SPF animal laboratory was provided by the Experimental Animal Center of Southwest Medical University as the experimental site with room temperature at 23°C–25°C, relative humidity 49%–51%, and 12 h light–dark cycle, and the feed was provided by the experimental animal center.

### 2.5 Acute Toxicity Study

In the acute toxicity study, 20 mice were randomly allocated to two groups (10 per sex) and fasted for 16 h before administration. The study protocol was conducted in compliance with the limited method (National Food Safety Standard Acute Oral Toxicity Test 2014). The DCL‐TP was prepared into a maximum concentration suspension of 125 mg/mL with 0.7% CMC. All animals were gavaged twice in 24 h, with an interval of 6 h. The volume of gavage was 0.4 mL/10 g bwt, and the total dose was 10 g/kg bwt. Clinical signs of toxicity were closely monitored for 1, 3, and 6 h after administration, and all rats were monitored twice daily for 14 days. Body weights were recorded before administration and on days 2, 7, and 14. On day 15, mice were sacrificed by cervical dislocation and were subjected to macroscopic examination, then the pathological changes in major organs such as the liver, heart, spleen, kidney were recorded, and the organ coefficients were calculated. The organ coefficient was calculated, which was calculated by dividing the wet weight of the organ by the body weight and multiplying it by 100, and the total phenol toxicity was judged by combining the body weight dose conversion table of humans and animals and the toxicity grade dose classification table.

$$\begin{array}{c} \text{The organ coefficient}\;\left(\%\right)=\text{the}\;\mathrm{wet}\;\text{weight of the organ}\\ \;/\text{the body weight}\times 100\% \end{array}$$

### 2.6 Three Genotoxicity Toxicity Tests

#### 2.6.1 Ames Test

The Ames test was conducted in accordance with the National Food Safety Standard Bacterial reverse mutation test (National Standardization Administration Committee, China, GB 15193.4–2014) (National Food Safety Standard Bacterial Reversion Mutation Test 2014). According to the plate infiltration method, five dose levels were set as 50, 158, 500, 1158, and 5000 μg/plate, with three plates for each dose. The test substance was diluted step by step $\sqrt{10}$ times to the concentration of each dose group. At the same time, the negative control (NC) group, the DMSO as solvent control (SC) group, and the positive control group (Ames kit standard mutagen) were set up. Three parallel tests were carried out in each dose group in the presence and absence of the metabolism activation system S_9_ according to the kit instructions, and the distance between the test dose groups was adjusted from $\sqrt{10}$ times to 5 times. The plates were incubated for 48 h at 37°C. The number of colonies was counted. A positive result was determined where the TA97A, TA98, TA100, WP2uvrA (pKM101) revertant colony counts were greater than 2‐fold, and the TA1535 revertant colony counts were greater than 3‐fold than those of the SC. On the contrary, The result was negative and then the verification tests were carried out according to 5 times the dose interval.

#### 2.6.2 Mammal Erythrocytes Micronucleus Test

The study protocol was conducted in mice of both sexes in accordance with the National Food Safety Standard mammal erythrocytes micronucleus test (National Standardization Administration Committee, China, GB 15193.5–2014) (National Food Safety Standard Chromosomal aberration test of spermatogonia or spermatocytes in mice 2014). 50 KM mice, aged 7–10 weeks, 22–28 g, were randomly assigned to five groups (25 males and 25 females): three dose groups were set up, which were 5, 2.5, and 1.25 g/kg bwt, respectively; at the same time, the 0.7% CMC and the cyclophosphamide (CYP) as NC group and the positive control (PC) group. The mice were twice gavaged with 0.4 mL/10 g bwt by the 30 h test method with the 24‐h interval, then they were sacrificed 6 h after the last administration; the femoral bone marrow was taken, and the hemostatic forceps were used to extrude the bone marrow. After mixing with calf serum, the smear was fixed and stained with Giemsa. When the bone marrow samples of each animal were analyzed, at least 200 bone marrow red blood cells (RBCs) were observed, and the percentage of polychromatic erythrocytes (PCEs) to total RBCs was calculated. The 2000 PCEs of each mouse were counted under the oil lens, and the micronucleus rate was calculated by dividing the number of cells containing micronucleus by the number of PCEs and multiplying it by 1000.

$$\begin{array}{c} \text{Micronucleus rate}\;\left(\%\right)=\\ \;\text{the number of cells containing micronucleus}\\ \;/\text{the number of polychromatic erythrocytes}\times 1000\% \end{array}$$

#### 2.6.3 Mouse Spermatocyte Chromosome Aberration Test

The study protocol was conducted in male mice in accordance with the National Food Safety Standard Mouse spermatocyte chromosome aberration test (National Standardization Administration Committee, China, GB 15193.8–2014) (National Food Safety Standard Mouse Spermatocyte Chromosome Aberration Test 2014). 25 male KM mice, aged 7–10 weeks and 21–30 g, were randomly assigned to five groups (25 male): three dose groups were set up, which were 5, 2.5, and 1.25 g/kg bwt, respectively; at the same time, the 0.7% CMC and the cyclophosphamide (CYP) as the NC group and the PC group. The mice in the PC group were injected with cyclophosphamide intraperitoneally.

Each dose group was oral with DCL‐TP suspension once a day for consecutive 5 days. On the 13th day after the last oral gavage, the mice were sacrificed 4 h after intraperitoneal injection of colchicine 6 mg/kg·bwt. The testes on both sides were removed, with hypotonic solution treatment, then the seminiferous tubules were gently separated, and 1% trisodium citrate solution was blown and stood still. The testes were taken, extracted seminiferous tubules then blowing and standing, fixed 2 times with methanol, centrifuged, sucked removed the upper clear liquid, then dropped on the ice water slide, dried naturally, and stained with Giemsa. When the metaphase cells were observed under an oil microscope, 100 cells per animal, 500 cells in each group, the abnormality and number of chromosome structural aberrations were counted.

### 2.7 28‐Day Oral Toxicity Study

The 28‐day oral toxicity study experimental design, 40 SD rats were randomly allocated to four groups (20 per sex). The study protocol was conducted in rats of both sexes in accordance with the National Food Safety Standard 28‐day oral toxicity test (National Standardization Administration Committee, China, GB 15193.22–2014) (National Food Safety Standard 28‐Day Oral Toxicity Test 2014). Doses of the DCL‐TP (0.625, 1.25, and 2.5 g/kg bwt) and 0.7% CMC (used as control) were administered by oral gavage once daily for 28 consecutive days.

During the experiments, all rats were allowed to water freely. The behaviors, general activities, signs and symptoms of toxicity, and mortality of the rats were observed regularly every day. The body weight of rats and the total food intake per cage which were measured every Thursday and Sunday were expressed by the total food utilization rate per cage, which was calculated by dividing the total weight gain per cage per week by the total weekly feed intake per cage and multiplying it by 100.

$$\begin{array}{c} \text{Total food utilization rate}\;\left(\%\right)\\ \;=\text{total weight gain}\;\mathrm{per}\;\text{cage}\;\mathrm{per}\;\text{week}\;\left(g\right)\\ \;/\text{the total weekly feed intake}\;\mathrm{per}\;\text{cage}\;\left(g\right)\times 100\% \end{array}$$

At the end of the experiment, urine routine using an Automatic Urine Analyzer (Model Mindray AVE752 Aiwei Technology Co. Ltd., Chin): urine glucose (GLU), urine protein (PRO), urine occult blood (BLD), uric acid alkalinity (pH), and urine specific gravity (SG) were measured in 12 h urine of rats. During the 16 h fasting but allowed to drinking, the rats were anesthetized. Blood samples were taken from the abdominal aorta to determine hematological indicators using an Automatic Haemotology Analyzer (Model MindrayBC‐680, Shenzhen Mindray Biomedical Co. Ltd., Chin): RBC, white blood cell (WBC), hemoglobin (HGB), neutrophil percentage (NEU‐R), lymphocyte percentage (LYM‐R), platelet (PLT), hematocrit (HCT), and blood biochemical indicators using an Automatic Biochemical Analyzer (Model MindrayBS‐2200 M, Shenzhen Mindray Biomedical Co. Ltd., China): alanine aminotransferase (ALT), aspartate aminotransferase (AST), urea (Urea), creatinine (Crea), blood glucose (Glu), total cholesterol (TC), triglyceride (TG), and uric acid (UA). Afterward, the changes in main organs such as the liver, heart, spleen, and kidney were recorded, and the organ coefficient was calculated by dividing the wet weight of the organ by the body weight and multiplying it by 100, and then the liver and kidney were fixed in 4% paraformaldehyde for HE staining, and the pathological changes were recorded.

$$\begin{array}{c} \text{The organ coefficient}\;\left(\%\right)=\text{the}\;\mathrm{wet}\;\text{weight of the organ}\\ \;/\text{the body weight}\times 100\% \end{array}$$

### 2.8 Statistical Analyses

All values obtained in this research were expressed as in the mean ± SD (standard error of the mean). The measurement data accord with the normal distribution, and the homogeneity of variance was analyzed by one‐way analysis of variance (ANOVA). The LSD test was used for post hoc multiple comparisons. Independent sample T‐test was used for comparison between the two samples. Nonparametric (Mann–Whitney U) rank sum test or binomial distribution test was used for unqualified data. The probability value results with (*p* < 0.05) were considered significant differences.

## 3 Result

### 3.1 The Results of Single‐Factor Examination and Orthogonal Experiment

**FIGURE 1:**
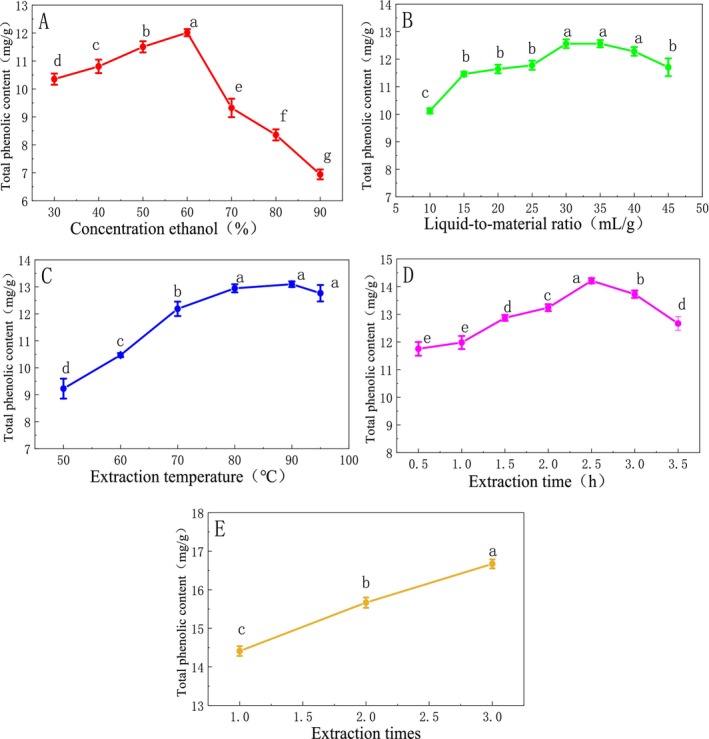
Effects of different parameters of heating reflux method on extraction amount of total phenols (different letters indicate significant differences with statistical significance, *p* < 0.05).

In Figure 1A, the trend of DCL‐TP extraction was first increased and then decreased. When the ethanol concentration was 60%, the total phenol extraction was the highest. In Figure 1B, the extraction amount of DCL‐TP increases first and then tends to be stable and last decreases in the range of 10–45 mL/g. At 35 mL/g, the extraction amount of DCL‐TP is the highest. There is no significant difference in the extraction amount of DCL‐TP between 30 and 40 mL/g (*p* > 0.05). The extraction temperature can be seen from Figure 1C, the trend of DCL‐TP extraction was first increased and then decreased in the range of 50°C–95°C. The total phenol extraction amount is the highest at 90°C (*p* > 0.05). The extraction time is shown in Figure 1D. The extraction amount of DCL‐TP increased first and then decreased in the range of 0.5–3.5 h, and DCL‐TP was the highest at 2.5 h. The effect of extraction times in Figure 1E, it can be seen that the extraction amount of DCL‐TP increased gradually with the increase of extraction times. The increase of extraction times can improve the amount of DCL‐TP.

Based on the single‐factor experiment, the extraction of DCL‐TP was carried out according to the L_18_(3^7^) orthogonal test design. The results are shown in Table 3, and the variance analysis is shown in Table 4.

**TABLE 3:** Orthogonal experimental design and results.

Test number	A (%)	B (mL/g)	C (°C)	D (h)	E	Extraction amount
	Concentration ethanol	Liquid‐to‐material ratio	Temperature	Time	Times	(mg/g)
1	1	1	1	1	1	13.53
2	1	2	2	2	2	16.73
3	1	3	3	3	3	18.17
4	2	1	1	2	2	14.60
5	2	2	2	3	3	17.07
6	2	3	3	1	1	14.02
7	3	1	2	1	3	15.49
8	3	2	3	2	1	12.96
9	3	3	1	3	2	14.23
10	1	1	3	3	2	17.07
11	1	2	1	1	3	16.09
12	1	3	2	2	1	15.59
13	2	1	2	3	1	14.44
14	2	2	3	1	2	16.06
15	2	3	1	2	3	15.54
16	3	1	3	2	3	16.50
17	3	2	1	3	1	12.00
18	3	3	2	1	2	15.65
K1	16.19	15.27	14.33	15.14	13.76	/
K2	15.29	15.15	15.83	15.32	15.72	/
K3	14.47	15.53	15.80	15.50	16.48	/
R	1.72	0.38	1.5	0.36	2.72	/

**TABLE 4:** Variance analysis of orthogonal experiment.

Factor	SST	DF	MS	F	*p*
A	8.935	2	4.468	40.160	< 0.05
B	0.457	2	0.229	2.054	> 0.05
C	8.774	2	4.387	39.437	< 0.05
D	0.382	2	0.191	1.715	> 0.05
E	23.667	2	11.834	106.374	< 0.05
Error	0.779	7	0.111	1	

*Note:* F0.05(2,7) = 4.74.

The heating reflux extraction process of DCL‐TP was optimized by the orthogonal experiment. The optimum extraction process of heating reflux was as follows: ethanol concentration 50%, liquid–solid ratio 30 mL/g, temperature 90°C, time 120 min, 2 times. The average extraction amount of DCL‐TP was 15.63 mg/g, and RSD was 1.15%.

### 3.2 Purification Parameters of DCL‐TP

**FIGURE 2:**
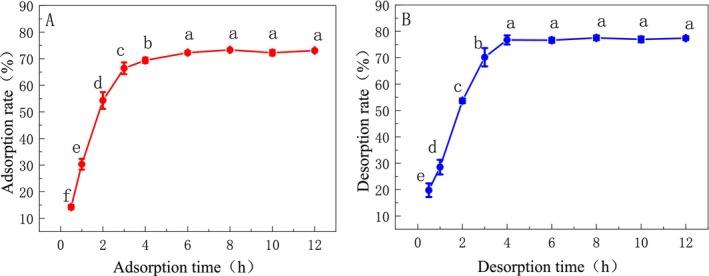
Static adsorption and desorption curve of HPD‐600 resin (different letters indicate significant differences with statistical significance, *p* < 0.05).

The adsorption kinetics of HPD‐600 resin on DCL‐TP is shown in Figure 2A, which shows that the adsorption rate of HPD‐600 resin on DCL‐TP, the trend of DCL‐TP extraction, was first increased then stable and reaches adsorption equilibrium in 6 h; therefore, the selection of the static adsorption time is 6 h, and the rate of adsorption can be up to 72.27%. In Figure 2B, the desorption rate of HPD‐600 resin on DCL‐TP rises the fastest in 4 h, tends to stabilize after 4 h, and basically reaches the resolution equilibrium, so the selection of resolution time is 4 h, and the resolution rate can be up to 76.74%.

**FIGURE 3:**
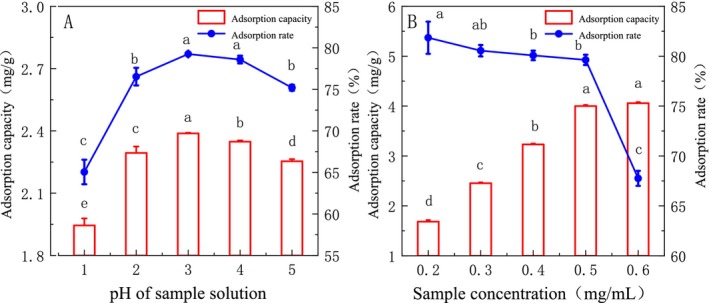
Effect of static parameters on the adsorption efficiency (different letters indicate significant differences with statistical significance, *p* < 0.05).

The effect of sample solution pH on static adsorption was shown in Figure 3A. With the increase of the pH of the sample solution, the adsorption capacity and adsorption rate increased first then decreased, and the adsorption capacity was the largest at pH = 3 (*p* < 0.05). The effect of sample concentration on static adsorption is shown in Figure 3B. With the increase of sample concentration, the adsorption capacity increased gradually, but when the sample concentration was 0.5–0.6 mg/mL, the adsorption capacity tended to be stable, and the adsorption rate decreased significantly with the increase of concentration (*p* < 0.05). To avoid excessive leakage and waste of raw materials, the optimum sample concentration was 0.5 mg/mL.

**FIGURE 4:**
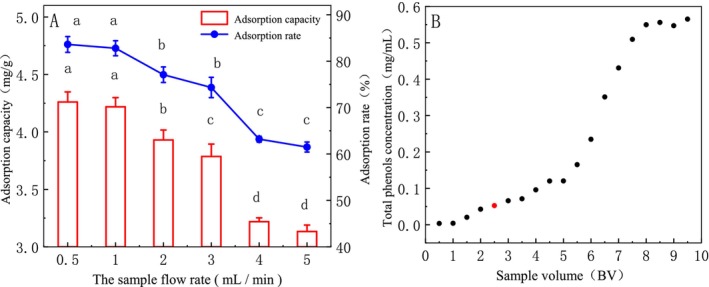
Effect of dynamic loading parameters on the adsorption efficiency (different letters indicate significant differences with statistical significance, *p* < 0.05).

The dynamic parameters (sample flow rate) in Figure 4A show that the adsorption capacity and adsorption rate of DCL‐TP decreased with the increase of sample flow rate but in the range of 0.5–1 mL/min (*p* > 0.05). The effect of the sample volume on the dynamic adsorption effect is shown in Figure 4B, which sees the total phenol concentration tends to be stable after 8BV. When 2.5 BV, the total phenol concentration is close to 1/10 of the concentration of the sample solution, indicating that the HPD‐600 resin has basically reached the adsorption saturation of the total phenol solution. To save the material cost, 2.5 BV is selected as the best sample volume.

**FIGURE 5:**
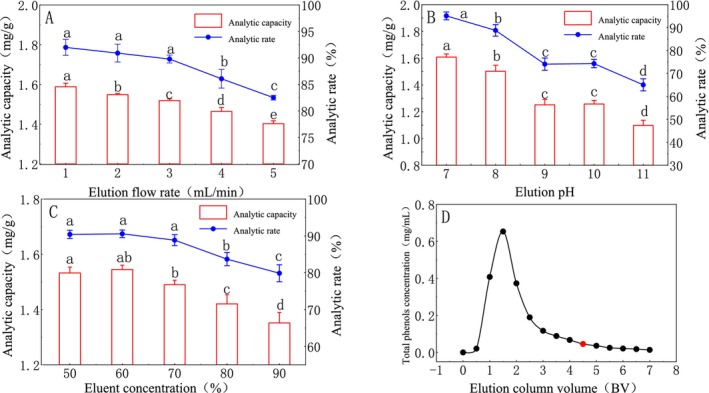
Effect of desorption parameters on the desorption efficiency (different letters indicate significant differences with statistical significance, *p* < 0.05).

The results of elution flow rate are shown in Figure 5A, with the increase in elution flow rate, the desorption amount decreased significantly (*p* < 0.05), and the optimal resolution flow rate was 1 mL/min. The elution pH is shown in Figure 5B. The desorption amount and rate decreased significantly with the increase of pH (*p* < 0.05); therefore, the elution pH was selected as 7. According to Figure 5C, the desorption amount and resolution rate are the highest when the eluent concentration is 60% (*p* < 0.05). In Figure 5D, when the effluent was 1.5 BV, the total phenol concentration reached the maximum, and then gradually decreased. When the effluent was 7 BV, the total phenol was basically resolved. However, considering the cost and time of the eluent, the elution efficiency was improved, so 4.5BV was selected as the elution volume.

According to the results of the above purification parameters, the optimum process for the purification of DCL‐TP by HPD‐600 resin was determined as follows: the concentration of sample solution was 0.5 mg/mL, the pH of sample solution was 3, the sample volume was 2.5 BV, the sample flow rate was 1 mL/min, the concentration of ethanol elution was 60%, the elution pH was 7, the elution flow rate was 1 mL/min, and 4.5 BV eluent was collected. Three batches of verification tests were carried out. The average purity of DCL‐TP was 26.40%, RSD was 1.7%, and the purity of DCL‐TP increased from 12.51% to 26.40%. It shows that the purification process is stable and reasonable.

### 3.3 Identification of DCL‐TP

**FIGURE 6:**
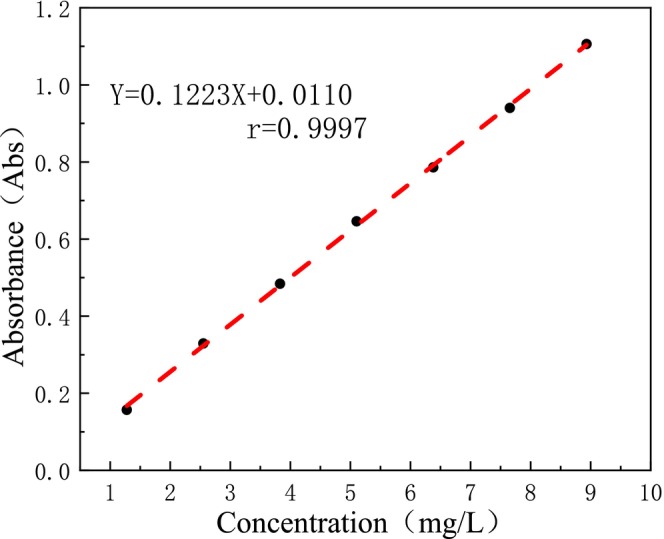
UV absorption profiles of reference substance and test sample.

**FIGURE 7:**
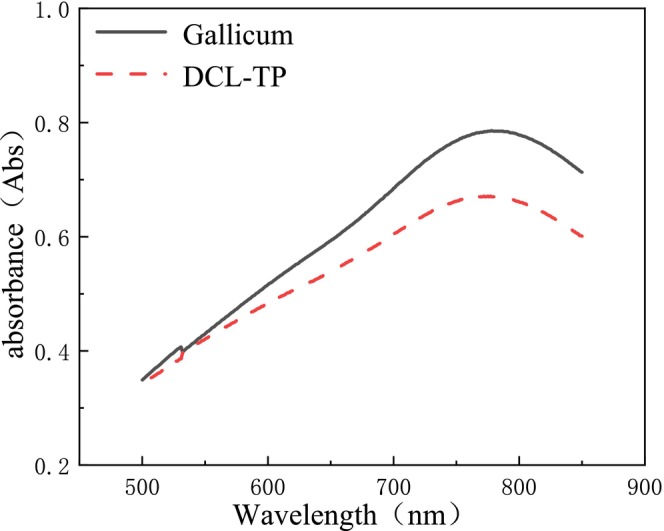
Gallic acid standard curve.

Zhang, Shi, and Yang (2017) explored the optimum conditions for the determination of DCL‐TP content by the Folin‐Ciocalteu method: the color reaction was carried out at 40°C for 20 min, and the absorbance was measured at 778 nm (Figure 6). Under this condition, the linear equation was: Y = 0.1223X + 0.0110, *r* = 0.9997(Figure 7). When the concentration of gallic acid was in the range of 1.276–8.932 mg/L, there was a good linear relationship between absorbance and concentration. Zhang et al. (2016) optimized the determination conditions of the Folin‐Ciocalteu colorimetric method, and determined the content of DCL‐TP was 15.79 mg/g (calculated by gallic acid).

In the precision test, the absorbance values were 0.644, 0.644, 0.646, 0.646, 0.646, 0.646, and 0.644. The average value was 0.645, and the RSD value was 0.17% (*n* = 6), indicating that the precision of the instrument was good. In the repeatability test, the results of DCL‐TP extraction were 15.85, 15.75, 16.29, 15.82, 15.46, and 15.60 mg/g, the average value was 15.79 mg/g, and the RSD value was 1.78% (*n* = 6), indicating that the method had good repeatability. In the stability test, the extract was measured every 15 min. The results were 15.85, 15.95, 16.05, 16.18, 16.28, 16.54, 16.49, 16.54 mg/g, the average value was 16.19 mg/g, and the RSD value was 1.61% (*n* = 7), indicating that the test solution was stable within 90 min. The recovery test results are shown in Table 5, with an average value of 97.61% and RSD value of 2.30% (*n* = 6), indicating that the recovery rate of this method is good.

**TABLE 5:** Results of sample adding recovery.

Sample volume (g)	Original volume (mg)	Adding volume (mg)	Measured volume (mg)	Recovery rate (%)	Mean (%)	RSD (%)
0.5011	7.83	7.82	15.64	99.82	97.61	2.30
0.5044	7.88	7.89	15.64	98.28		
0.5019	7.84	7.85	15.33	95.37		
0.5028	7.86	7.80	15.38	96.46		
0.5087	7.95	7.96	15.54	95.28		
0.5051	7.89	7.91	15.84	100.47		

### 3.4 Acute Toxicity Study

In the acute toxicity study, no animals died in the 10 g/kg bwt dosing group. During the 14‐day observation period, no symptoms of toxicity and mortality were observed in the animals. Therefore, the LD_50_ of the mice was > 10 g/kg bwt. According to Table 6, the dose of 10 g/kg bwt in mice is equivalent to the dose of 7 g/kg bwt in 200 g rats, which belongs to the actual nontoxic grade according to the standard of Table 7.

**TABLE 6:** Dose conversion coefficient of animal and human body weight per kilogram.

Conversion coefficient W			A kind of human animal						
			Mouse 0.02 kg	Rat 0.2 kg	Guinea pig 0.4 kg	Rabbit 1.5 kg	Cat 2.0 kg	Dog 12 kg	Adult 60 kg
B kind of human animal	Mouse	0.02 kg	1.0	1.6	1.4	2.7	3.2	4.8	9.01
	Rat	0.2 kg	0.7	1.0	1.14	1.88	2.3	3.6	6.25
	Guinea pig	0.4 kg	0.61	0.87	1.0	1.65	2.05	3.0	5.55
	Rabbit	1.5 kg	0.37	0.52	0.6	1.0	1.23	1.76	2.30
	Cat	2.0 kg	0.30	0.42	0.48	0.81	1.0	1.44	2.70
	Dog	12 kg	0.21	0.28	0.34	0.56	0.07	1.0	1.88
	Adult	60 kg	0.11	0.16	0.18	0.30	0.37	0.53	1.0

**TABLE 7:** Classification standards of acute toxicity dose.

Standard	Oral LD50 of rats (mg/kg)	Equivalent to the lethal dose of human beings	
		(mg/kg)	(g/person)
Extremely toxicity	< 1	Slightly taste	0.05
Poisonous	1 ~ 50	500 ~ 4000	0.5
Medium toxicity	51 ~ 500	4000 ~ 30,000	5
Low toxicity	501 ~ 5000	30,000 ~ 250,000	50
Nontoxicity	> 5000	250,000 ~ 500,000	500

During the experiment, the weight of mice in each group increased to a certain extent. The weight gain of mice is shown in Table 8. The weight gain of male mice was higher than that of female mice. The organ coefficients of mice are shown in Table 9. The organ coefficients of the liver, heart, and spleen of male mice were not statistically different from those of female mice (*p* > 0.05), and the organ coefficients of male kidney were statistically different from those of female mice (*p* < 0.05), which were in line with the normal growth and development of mice. Similarly, no significant pathology was observed in all organs within the dose range of 10 g/kg bwt.

**TABLE 8:** Body weight changes of mice in acute toxicity study.

Groups	D_0_ weight (g)	D_7_ weight (g)	D_14_ weight (g)	Total gain (g)
Female	20.9 ± 1.1	26. ± 1.3	35.9 ± 2.1	15.0 ± 1.8
Male	21.6 ± 2.0	28.8 ± 2.5	41.5 ± 3.3	19.8 ± 3.3

**TABLE 9:** Percentage coefficient of organs of mice in acute toxicity study.

Groups	Liver (%)	Cardiac (%)	Spleen (%)	Kidney (%)
Female	5.10 ± 0.25	0.51 ± 0.06	0.39 ± 0.07	1.13 ± 0.09
Male	5.30 ± 0.37	0.51 ± 0.05	0.35 ± 0.04	1.44 ± 0.20*

*Note:* Comparison with the female group, *p < 0.05, **p < 0.01.

### 3.5 Bacterial Reverse Mutation Test

The number of TA97a, TA98, TA100, WP2uvrA (pKM101), and TA1535 strains is recorded in Tables 10, 11. The number of reverse colonies in each PC group was more than 2 times that of the NC group, and there was a significant difference (*p* < 0.01), indicating that the test conditions were stable and the results were credible. The average number of reverse colonies in each dose group of DCL‐TP was not more than 2 times that of TA97A, TA98 and TA100 WP2uvrA (pKM101) SC groups, and not more than 3 times that of TA1535 SC group (*p* > 0.05). In the case of different concentrations of DCL‐TP with or without S9, the number of reverse colonies in each dose group of TA97a, TA98, and TA1535 strains was not significantly different from that in the SC group (*p* > 0.05), and there was no dose–response relationship. However, The number of reverse colonies of TA100 and WP2uvrA (pKM101) strains in the high‐dose group (5000 μg/plat) was significantly lower than that in the SC group (*p* < 0.05).

**TABLE 10:** Results of the Ames test (first).

Groups	Dose	TA97a		TA98		TA100		WP2uvrA (pKM101)		TA1535	
	(μg/plat)	‐S9	+S9	‐S9	+S9	‐S9	+S9	‐S9	+S9	‐S9	+S9
Control	0	110.7 ± 14.0	104.0 ± 12.8	26.3 ± 3.2	32.3 ± 6.7	126.7 ± 4.6	152.0 ± 4.0	126.3 ± 22.9	140.0 ± 14.0	13.0 ± 3.0	12.7 ± 1.2
DMSO	0	112.7 ± 16.3	95.3 ± 7.6	25.3 ± 2.1	33.3 ± 8.4	116.3 ± 8.5	150.0 ± 8.7	130.0 ± 6.0	140.3 ± 11.0	11.0 ± 1.0	12.0 ± 1.0
DCL‐TP	50	139.0 ± 20.8	105.3 ± 5.8	34.0 ± 4.4	43.3 ± 8.0	126.7 ± 10.1	158.0 ± 10.4	138.3 ± 6.8	151.0 ± 20.1	12.3 ± 0.6	13.0 ± 1.7
	158	131.3 ± 32	143.3 ± 33.0	32.3 ± 8.0	44.3 ± 3.5	147.3 ± 13.3	166.7 ± 17.5	136.3 ± 10.1	157.0 ± 9.8	14.3 ± 2.9	15.3 ± 1.2
	500	129.3 ± 18.5	123.7 ± 22.8	26.7 ± 5.8	37.7 ± 7.2	126.0 ± 9.5	179.3 ± 12.1	141.0 ± 18.2	148.0 ± 17.4	13.3 ± 2.9	14.3 ± 2.9
	1580	141.3 ± 16.3	108.3 ± 12.5	28.7 ± 9.6	38.0 ± 9.2	122.0 ± 14.0	151.0 ± 7.5	131.0 ± 16.6	138.0 ± 13.0	16.0 ± 1.0	14.7 ± 2.5
	5000	128.0 ± 28.8	115.3 ± 14.4	29.3 ± 2.1	37.7 ± 6.4	89.7 ± 8.7^#^	120.3 ± 8.1^#^	103.7 ± 25.7^#^	121.3 ± 18.9^#^	12.7 ± 2.3	13.3 ± 0.6
Dexon	50	1891.3 ± 167.7^**^		976.7 ± 122.2**							
2‐aminofluorene	20		1549.0 ± 165.2**		3993.0 ± 321.1**		1068.3 ± 328.1**				
Methyl methanesulfonate	1 μL					881.7 ± 46.8**		935.7 ± 213.0**			
2‐Aminoanthracene	2								450.7 ± 118.0**		584.0 ± 122.9**
Sodium azide	1.5									743.7 ± 95.5**	

*Note:* Compared with the negative group, *p < 0.05, **p < 0.01; compared with the solvent group, ^#^ *p* < 0.05, ^##^ *p* < 0.01.

**TABLE 11:** Results of the Ames test (verification).

Groups	Dose	TA97a		TA98		TA100		WP2uvrA (pKM101)		TA1535	
	(μg/ plat)	‐S9	+S9	‐S9	+S9	‐S9	+S9	‐S9	+S9	‐S9	+S9
Control	0	116.3 ± 9.7	116.7 ± 9.5	23.7 ± 2.3	33.3 ± 4.5	138.0 ± 9.2	146.0 ± 15.6	143.7 ± 20.7	152.0 ± 19.1	11.7 ± 2.3	13.0 ± 2.0
DMSO	0	112.0 ± 10.6	111.3 ± 10.3	23.3 ± 2.3	27.0 ± 1.0	134.0 ± 7.2	143.3 ± 15.3	137.7 ± 13.1	145.7 ± 17.8	10.3 ± 2.1	11.0 ± 1.0
DCL‐TP	8	127.3 ± 13.3	113.7 ± 10.6	23.7 ± 2.1	26.7 ± 3.1	136.3 ± 5.7	149.3 ± 16.2	140.7 ± 8.1	140.0 ± 19.1	11.3 ± 1.5	12.7 ± 2.1
	40	120.0 ± 5.3	138.7 ± 12.2	25.7 ± 2.1	34.7 ± 11.9	156.0 ± 21.2	168.7 ± 21.5	143.3 ± 15.3	157.3 ± 15.1	13.7 ± 2.5	13.3 ± 0.6
	200	136.3 ± 11.0	143.7 ± 4.5	24.7 ± 5.0	36.3 ± 8.5	148.7 ± 4.7	168.0 ± 17.1	159.3 ± 24.2	166.0 ± 28.0	12.7 ± 1.2	13.3 ± 3.2
	1000	152.7 ± 13.3	148.0 ± 23.1	30.3 ± 3.2	36.7 ± 3.8	134.7 ± 9.5	148.7 ± 7.6	146.7 ± 18.0	148.0 ± 18.1	15.0 ± 2.0	16.3 ± 2.3
	5000	139.3 ± 12.1	135.0 ± 7.9	33.0 ± 5.2	34.7 ± 4.9	115.7 ± 10.0#	122.3 ± 8.3#	114.0 ± 15.7#	124.0 ± 10.0#	13.3 ± 2.3	15.0 ± 2.0
Dexon	50	2168.0 ± 373.9**		1107.3 ± 63.1**							
2‐Aminofluorene	20		1483.7 ± 99.7**		4219.3 ± 114.3**		1226.3 ± 229.1**				
Methyl methanesulfonate	1 μL					881.7 ± 46.8**		1061.7 ± 102.7**			
2‐Aminoanthracene	2								492.0 ± 65.2**		529.0 ± 62.3**
Sodium azide	1.5									938.7 ± 105.3**	

*Note:* Compared with the negative group, *p < 0.05, **p < 0.01; compared with the solvent group, #*p* < 0.05, ##*p* < 0.01.

### 3.6 Mammalian Erythrocyte Micronucleus Test

The PCE/RBC values of female and male mice treated with DCL‐TP at 1.25, 2.5, and 5 g/kg bwt, dose groups were 20% higher than the NC group. The effect of DCL‐TP on the micronucleus rate of mouse bone marrow is shown in Table 12. There was no significant difference in the micronucleus rate between the male and female dose groups treated with DCL‐TP and the NC group (*p* > 0.05). The micronucleus rate of male and female PC groups treated with CYP was significantly higher than that of the NC group (*p* < 0.01), suggesting that the DCL‐TP has no mutagenic effect on the micronucleus rate of mouse bone marrow cells.

**TABLE 12:** Micronucleus test of bone marrow erythrocytes in mice.

Groups	Contains micronucleus PCE (*N*)	PCE counts	Micronucleus frequency	PCE/RBC
		(*N*)	(‰)	(%)
**Female**				
Control	29	10,000	2.9 ± 0.7	53.3 ± 0.8
Cyclophosphamide	36	10,000	3.6 ± 0.4	51.4 ± 0.4
1.25 g/kg·bwt	31	10,000	3.1 ± 0.2	51.8 ± 0.6
2.50 g/kg·bwt	37	10,000	3.7 ± 0.6	50.2 ± 0.6
5 g/kg·bwt	198	10,000	19.8 ± 0.9**	48.4 ± 0.4
**Male**				
Control	32	10,000	3.2 ± 0.8	52.9 ± 0.8
Cyclophosphamide	35	10,000	3.5 ± 0.5	52.0 ± 0.5
1.25 g/kg·bwt	31	10,000	3.1 ± 0.2	52.0 ± 0.8
2.50 g/kg·bwt	36	10,000	3.6 ± 0.7	51.3 ± 0.3
5 g/kg·bwt	196	10,000	19.6 ± 1.0**	48.2 ± 0.6

*Note:* Comparison with the negative group, *p < 0.05, **p < 0.01.

### 3.7 Mouse Spermatocyte Chromosome Aberration Test

The effect of DCL‐TP on the chromosome aberration test of spermatocytes in mice is shown in Table 13. There was no significant difference in the rate of aberration cells between the DCL‐TP‐treated groups and the negative group (*p* > 0.05). However, the rate of aberration cells in the PC group treated (CYP) was significantly higher than that in the NC group (p < 0.01), which proved that DCL‐TP has not cause chromosome aberration of spermatocytes in mice in the dose range of 5 g/kg bwt.

**TABLE 13:** Chromosome aberration test of mouse spermatocyte.

Groups	Structural aberration		Monovalent		Distorted cell counts	Distortion cell frequency
	Fragment	Displace	Autosomal chromosome	Sex chromosome	(*N*)	(%)
Control	5	0	4	2	5	1
Cyclophosphamide	4	0	5	3	4	0.8
1.25 g/kg·bwt	5	0	6	2	5	1
2.50 g/kg·bwt	7	0	5	3	7	1.4
5 g/kg·bwt	37	2	43	9	39	7.8**

*Note:* Comparison with the negative group, *p < 0.05, **p < 0.01.

### 3.8 28‐Day Oral Toxicity Study

#### 3.8.1 Clinical Observations, Body Weight, and Mean Food Consumption

**FIGURE 8:**
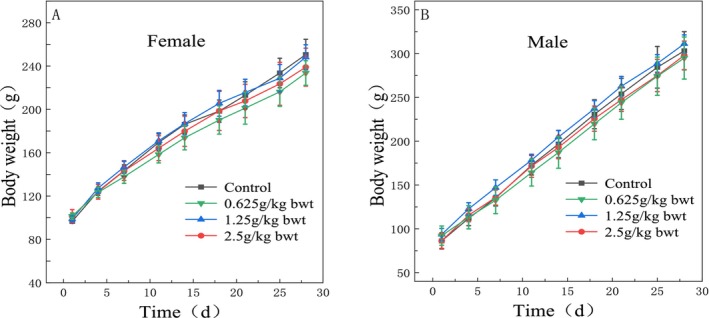
Effects of total phenols on body weight change in rats.

**FIGURE 9:**
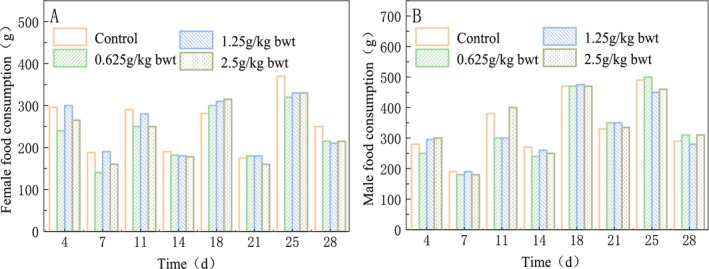
Effect of total phenols on food total consumption of rats.

During the dosing period of 28 days, no animal died and no abnormal behavior or overt signs of toxicity were observed in the rats of each dose group. Compared with the control group, there were no statistically significant differences in drinking water, eating, coat color, behavior, feces, urine excretion, body weight, total food consumption, and total food utilization in each group (*p* > 0.05) (Figures 8, 9, Table 14).

**TABLE 14:** Effect of total phenols on food total availability of rats.

Groups	Total utilization rate (%)				
	D_1_	D_14_	D_21_	D_28_	Mean
**Female**					
Control	48.3	44.6	29.4	30.3	37.7
0.625 g/kg bwt	47.8	41.7	28.5	30.3	36.2
1.25 g/kg bwt	48.9	43.5	29.4	30.2	37.7
2.5 g/kg bwt	49.2	42.8	29.5	28.6	36.7
**Male**					
Control	52.1	47.4	35.7	31.5	40.2
0.625 g/kg·bwt	47.0	50.6	34.6	31.5	39.0
1.25 g/kg·bwt	55.1	51.6	35.2	33.2	41.8
2.5 g/kg·bwt	51.6	44	33.8	32.9	39.1

#### 3.8.2 Hematological and Blood Biochemical

Results of the hematology analyses are shown in Supplementary Table 15. At the end of the experiment, there was no significant differences in hematological indexes between the female and male dose groups treated with DCL‐TP and the NC group (*p* > 0.05), which showed that DCL‐TP had no significant effect on the main hematological indexes of rats in the dose range of 2.5 g/kg bwt.

**TABLE 15:** Effects of total phenols on hematological parameters in rats.

Groups	WBC	NEU‐R	LYM‐R	RBC	HGB	HCT	PLT
	(10^9^/L)	(%)	(%)	(10^12^/L)	(g/L)	(L/L)	(10^9^/L)
**Female**							
Control	5.66 ± 0.52	16.25 ± 2.53	81.10 ± 2.65	6.48 ± 0.22	144.50 ± 3.11	0.47 ± 0.00	900.25 ± 51.68
0.625 g/kg bwt	4.24 ± 2.07	13.30 ± 2.77	82.38 ± 2.32	6.95 ± 0.44	146.50 ± 11.15	0.46 ± 0.004	808.50 ± 124.74
1.25 g/kg bwt	6.71 ± 1.66	18.03 ± 5.74	77.43 ± 5.82	6.98 ± 0.25	148.50 ± 2.38	0.47 ± 0.01	744.25 ± 112.47
2.5 g/kg bwt	5.16 ± 1.79	20.15 ± 11.79	74.70 ± 12.15	6.99 ± 0.60	147.50 ± 12.45	0.47 ± 0.04	873.50 ± 119.83
**Male**							
Control	6.39 ± 1.02	21.65 ± 3.30	74.75 ± 3.89	6.73 ± 0.27	141.75 ± 6.40	0.46 ± 0.03	846.25 ± 70.28
0.625 g/kg bwt	7.75 ± 0.64	15.55 ± 6.64	81.48 ± 6.47	7.02 ± 0.50	151.25 ± 4.27	0.48 ± 0.01	782.00 ± 152.84
1.25 g/kg bwt	5.33 ± 1.32	13.30 ± 1.24*	81.78 ± 2.49	7.30 ± 0.55	151.75 ± 5.50	0.48 ± 0.01	678.75 ± 305.75
2.5 g/kg bwt	7.43 ± 2.08	16.93 ± 5.58	78.53 ± 6.27	7.02 ± 0.43	148.00 ± 10.68	0.47 ± 0.03	731.25 ± 192.53

*Note:* Comparison with the negative group, *p < 0.05, **p < 0.01.

Results of the clinical chemistry analyses are shown in Table 16, and there were no significant difference in blood biochemical indexes between the female groups treated with DCL‐TP and the NC group (*p* > 0.05). With the exception of the Crea index in high‐dose males (5 g/kg bwt) being higher than that the controls (*p* < 0.05), there were no significant differences in other blood biochemical indexes results in the male groups and NC groups (*p* > 0.05). However, these differences were considered meaningless due to being within their corresponding normal ranges and the average difference was only 4.3 μmol/L (Xiao et al. 2021).

**TABLE 16:** Effects of total phenols on blood biochemical parameters in rats.

Groups	ALT	AST	Urea	UA	Crea	TC	TG	GLU
	(U/L)	(U/L)	(mmol/L)	(μmol/L)	(μmol/L)	(mmol/L)	(mmol/L)	(mmol/L)
**Female**								
Control	43.13 ± 16.36	171.30 ± 56.80	6.08 ± 2.12	153.58 ± 17.49	23.13 ± 11.70	1.81 ± 0.15	0.37 ± 0.14	6.26 ± 0.80
0.625 g/kg bwt	37.63 ± 14.74	138.03 ± 47.59	7.09 ± 1.25	142.48 ± 10.24	29.90 ± 10.62	2.04 ± 0.56	0.34 ± 0.17	6.16 ± 1.10
1.25 g/kg bwt	33.85 ± 11.66	120.95 ± 25.81	8.14 ± 1.11	139.85 ± 29.15	33.75 ± 4.71	1.92 ± 0.31	0.25 ± 0.04	6.58 ± 0.41
2.5 g/kg bwt	27.38 ± 1.85	116.73 ± 24.79	6.86 ± 1.27	148.55 ± 10.28	28.50 ± 3.65	1.77 ± 0.32	0.29 ± 0.08	7.28 ± 0.75
**Male**								
Control	41.60 ± 5.18	148.75 ± 41.50	6.36 ± 1.27	137.20 ± 31.71	24.85 ± 3.14	2.35 ± 0.33	0.69 ± 0.39	6.68 ± 0.80
0.625 g/kg bwt	38.28 ± 4.64	155.18 ± 28.88	5.86 ± 1.02	154.00 ± 36.71	21.70 ± 2.08	2.05 ± 0.60	0.50 ± 0.14	7.49 ± 1.29
1.25 g/kg bwt	43.48 ± 15.10	205.18 ± 45.03	6.91 ± 0.64	140.45 ± 30.62	22.73 ± 1.03	1.89 ± 0.38	0.60 ± 0.22	7.03 ± 1.02
2.5 g/kg bwt	37.20 ± 3.66	110.33 ± 9.18	6.19 ± 1.14	134.40 ± 11.37	29.15 ± 1.32*	2.16 ± 0.14	0.58 ± 0.14	6.97 ± 0.85

*Note:* Comparison with the negative group, *p < 0.05, **p < 0.01.

#### 3.8.3 Organ Coefficient and Macroscopic Findings

The main organ coefficients (liver, heart, spleen, and kidney) are shown in Table 17, at the end of the experiment, there were no significant differences in the main organ coefficients results in each dose group treated with DCL‐TP and control negative groups (*p* > 0.05), which indicated that DCL‐TP had no significant effect on the main organ coefficient of rats in the dose range of 2.5 g/kg bwt for 28 days. In the macroscopic examination, there were no macroscopic signs of pathology in all groups.

**TABLE 17:** Effect of total phenols on organ coefficient in rats.

Groups	Liver coefficient (%)	Heart coefficient (%)	Spleen coefficient (%)	Kidney coefficient coefficients (%)
**Female**				
Control	3.48 ± 0.17	0.41 ± 0.04	0.26 ± 0.01	0.94 ± 0.04
0.625 g/kg bwt	3.14 ± 0.19	0.40 ± 0.03	0.22 ± 0.02	0.83 ± 0.04
1.25 g/kg bwt	3.38 ± 0.60	0.40 ± 0.04	0.24 ± 0.06	0.83 ± 0.15
2.5 g/kg bwt	3.28 ± 0.31	0.37 ± 0.03	0.23 ± 0.01	0.83 ± 0.06
**Male**				
Control	3.08 ± 0.19	0.39 ± 0.04	0.21 ± 0.01	0.78 ± 0.01
0.625 g/kg bwt	3.19 ± 0.25	0.43 ± 0.07	0.24 ± 0.03	0.85 ± 0.07
1.25 g/kg bwt	3.03 ± 0.11	0.39 ± 0.04	0.20 ± 0.03	0.81 ± 0.06
2.5 g/kg bwt	2.86 ± 0.20	0.40 ± 0.04	0.21 ± 0.02	0.80 ± 0.03

*Note:* Comparison with the negative group, *p < 0.05, **p < 0.01.

#### 3.8.4 Urinalysis

The urinalysis results (urine glucose, urine protein, urine occult blood, pH, and urine SG) are shown in Table 18, and there were no significant differences in the urinalysis results in the dose male and female with DCL‐TP and NC groups (*P* > 0.05), which showed DCL‐TP had no significant effect on the urine index in high dose of 2.5 g/kg bwt.

**TABLE 18:** Effects of total phenols on urinary parameters in rats.

Index	Degree	Female (*N* = 4)				Male (*N* = 4)			
		Dose (mg/kg bwt)				Dose (mg/kg bwt)			
		0	625	1250	2500	0	625	1250	2500
GLU	—	4	4	4	4	4	4	4	4
	+ −	0	0	0	0	0	0	0	0
	1+	0	0	0	0	0	0	0	0
	2+	0	0	0	0	0	0	0	0
PRO	—	0	4	4	4	0	0	4	4
	+ −	1	0	0	0	2	4	0	0
	1+	3	0	0	0	2	0	0	0
	2+	0	0	0	0	0	0	0	0
BLD	—	4	4	4	4	4	4	4	4
	+ −	0	0	0	0	0	0	0	0
	1+	0	0	0	0	0	0	0	0
	2+	0	0	0	0	0	0	0	0
SG	1.000	0	4	0	0	0	0	0	0
	1.005	0	0	4	4	0	4	0	0
	1.010	0	0	0	0	0	0	4	3
	1.015	4	0	0	0	4	0	0	1
	1.020	0	0	0	0	0	0	0	0
pH	6.5	0	0	0	0	0	0	0	0
	7.0	0	0	0	0	0	0	0	0
	7.5	3	4	4	4	4	3	4	4
	8.0	1	0	0	0	0	1	0	0

*Note:* Comparison with the negative group, *p < 0.05, **p < 0.01.

#### 3.8.5 Histopathology

Gross anatomical examination of the vital organs (liver, kidney, heart, lung, spleen, and testis) in the 28‐day oral toxicity study did not reveal any gross pathological lesions in color, morphology, and size.

**FIGURE 10:**
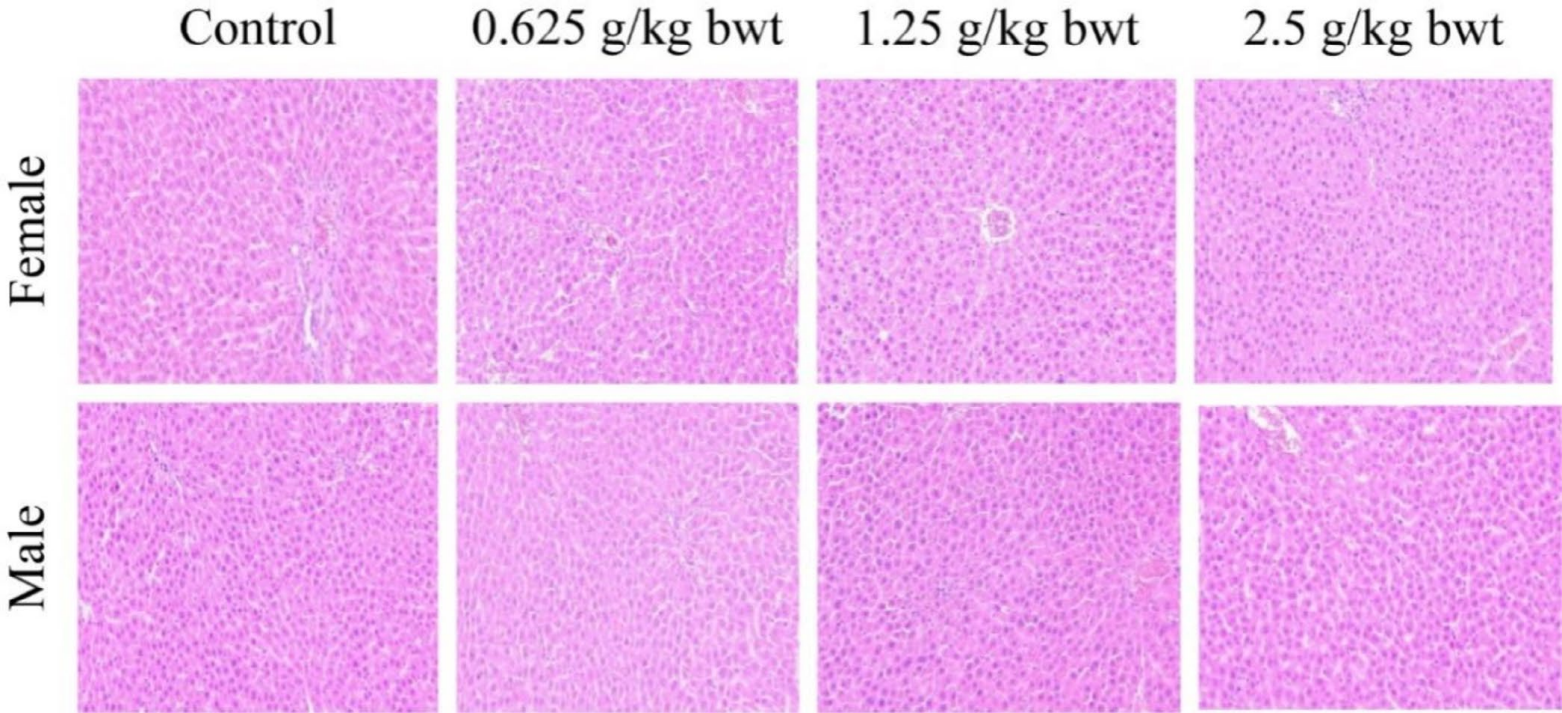
Results of hematoxylin–eosin staining in the liver 200×.

**FIGURE 11:**
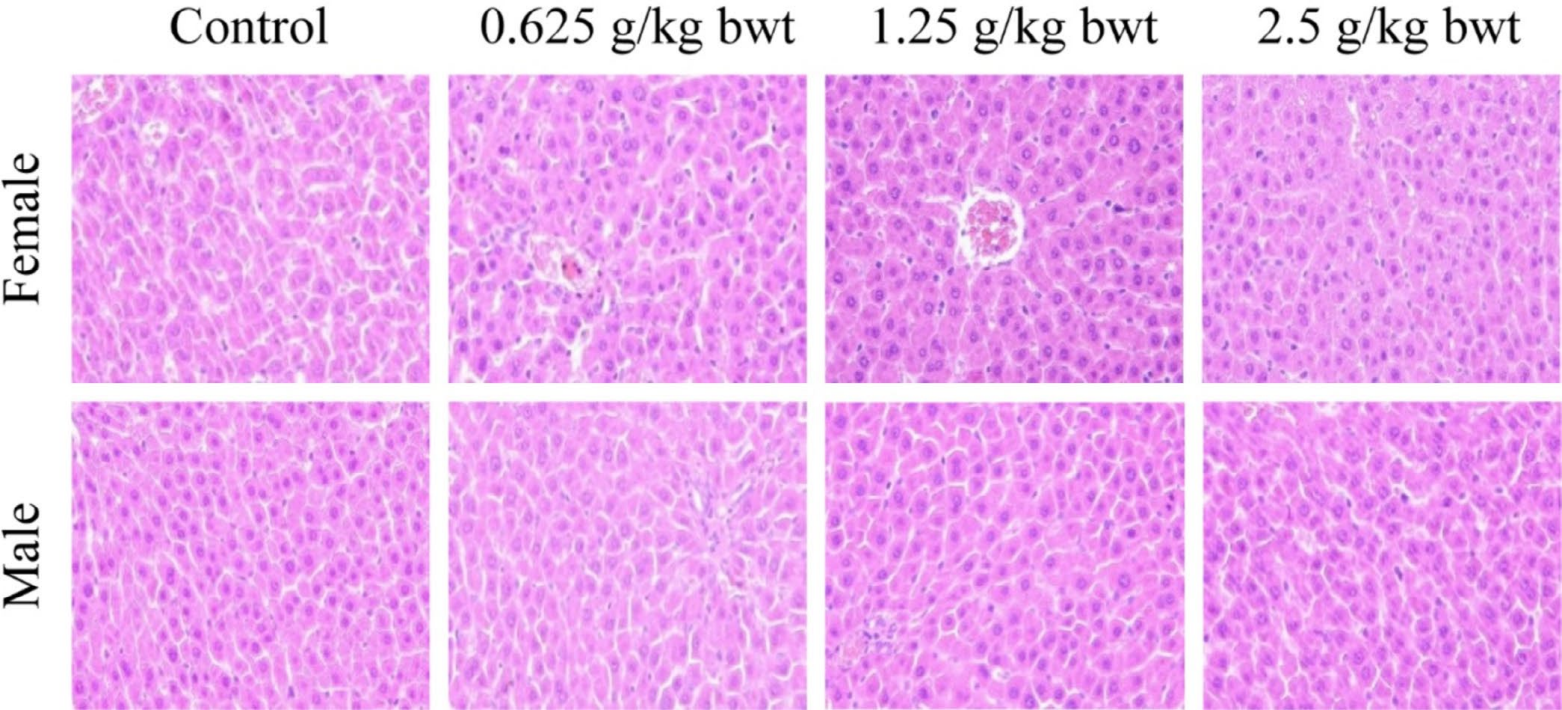
Results of hematoxylin–eosin staining in the liver 400×.

**FIGURE 12:**
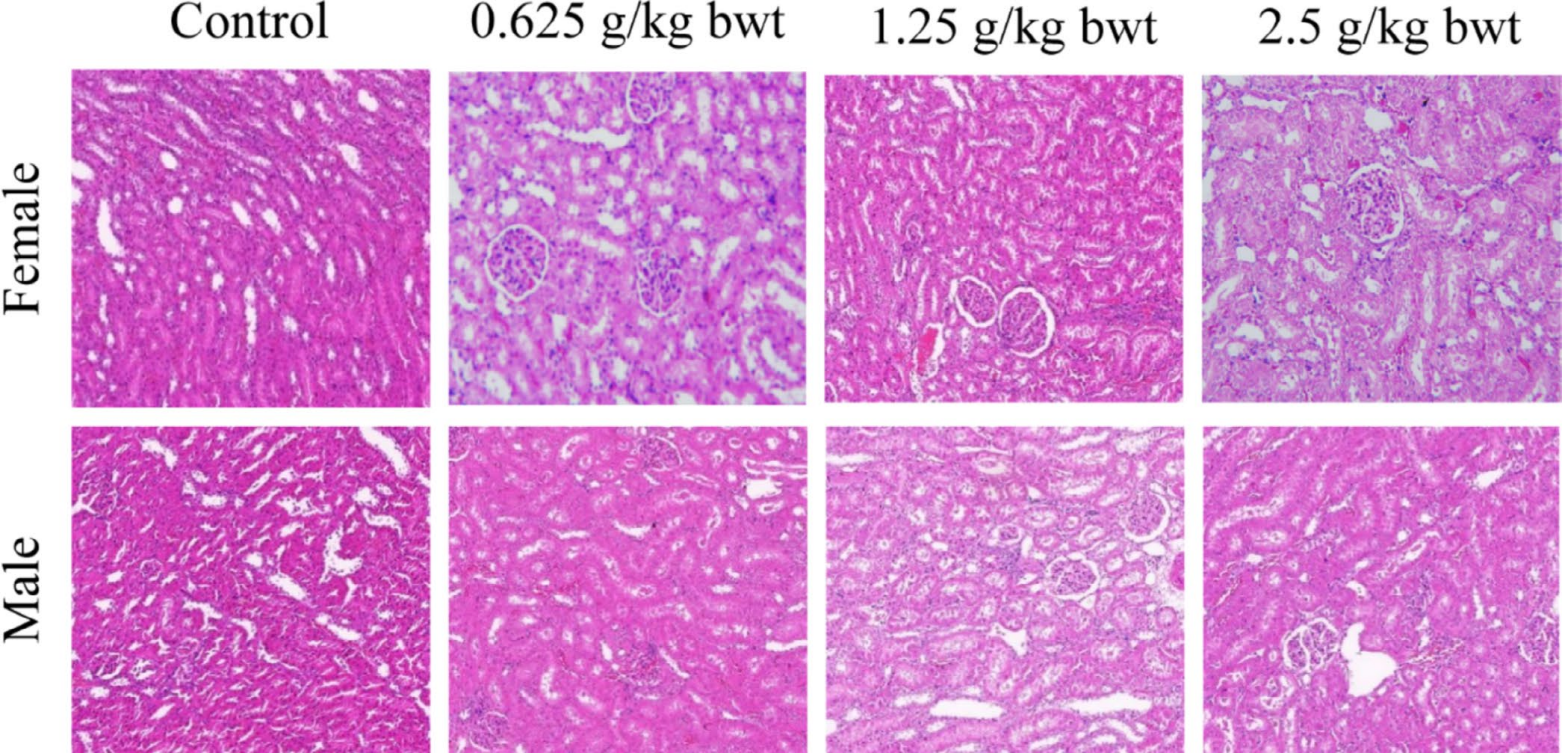
Results of hematoxylin–eosin staining in the kidney 200×.

**FIGURE 13:**
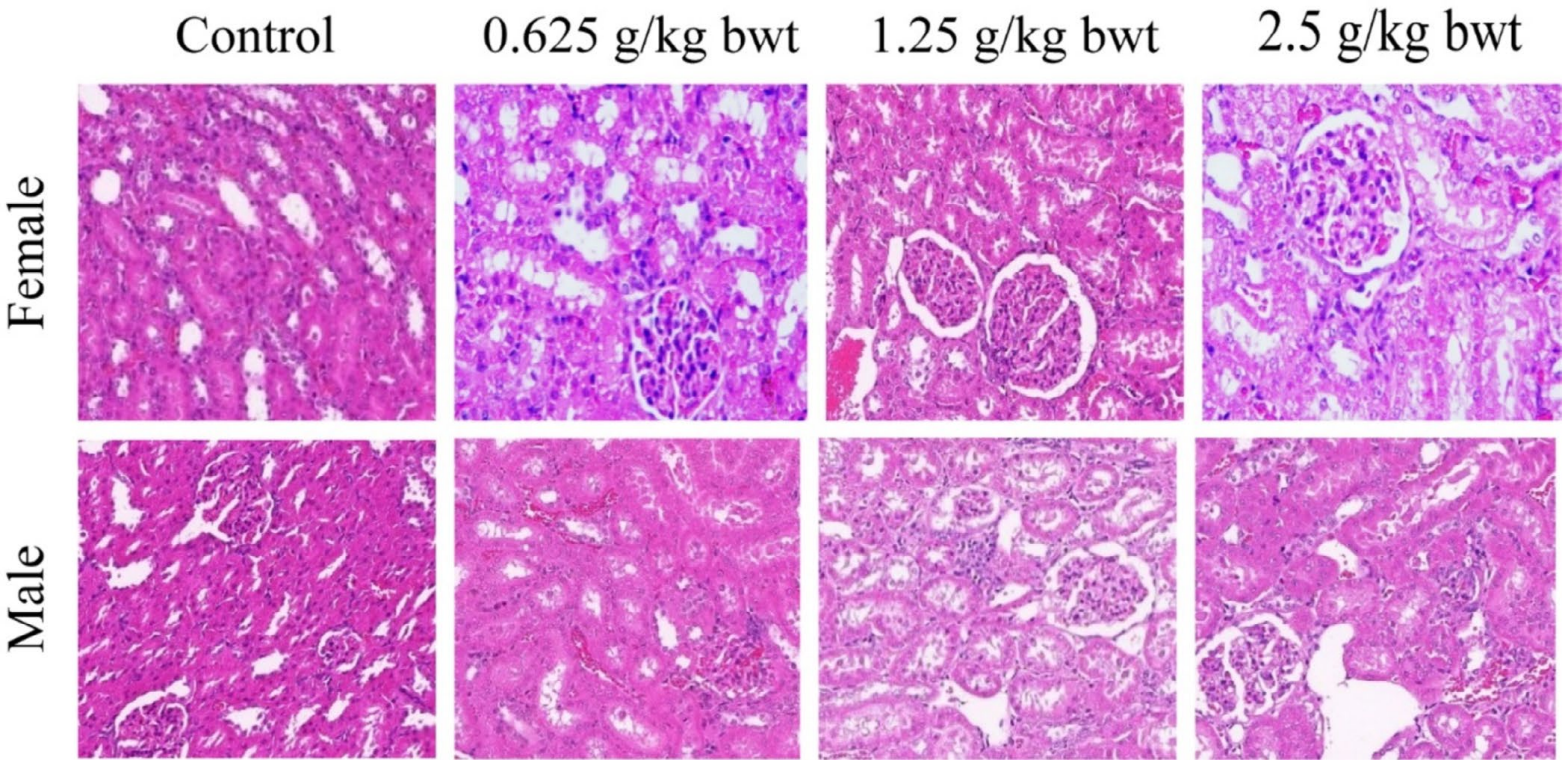
Results of hematoxylin–eosin staining in the kidney 400×.

The liver and kidney are important organs for the metabolism and transformation of total phenols in rats. Therefore, pathological tissue photographs of the liver from a female and a male for the control, high‐dose (2.5 g/kg bwt), mid‐dose (1.25 g/kg bwt), and low‐dose (0.625 g/kg bwt) groups are shown in Figures 10, 11. The tissue sections were stained with HE. then at Biomicroscope (Model Mindray CX40; Ningbo Shunyu Intelligent Technology Co. Ltd., China) liver cells radially arranged in an orderly manner, each part of the lobule is clearly visible, no obvious inflammatory cell infiltration, liver tissue structure is normal, liver cell nucleolus clear, no obvious lipid droplet accumulation in liver cells. The kidney showed normal morphological structure among the different dose levels, and controls and pathological tissue photographs are shown in Figures 12, 13. The structure of renal cortex and medulla tissue was normal when examined using a biomicroscope. No damage or lesion was found in the glomeruli and renal tubule, and there was no obvious inflammatory cell infiltration in the renal interstitium.

## 4 Discussion

DCL‐TP exhibits significant potential in antilipid oxidation food additives. Yet, the underlying edible safeties are still unclear. In this study, we successfully prepared a DCL‐TP concerning antioxidant properties. We further assessed the behavioral signs, organ coefficient of mice and body weight, poisoning death, and exercise activity of rats at the macroscopic findings. Furthermore, of note, from microcosmic findings, the safety of the DCL‐TP was revealed based on the analysis of spontaneous reverse colony number of TA97a, TA98, TA100, WP2uvrA (pKM101), TA1535 strains, micronucleus formation rate analysis of mouse bone marrow cells, chromosome aberration rate of mouse spermatocyte, hematological, blood biochemical and urine indexes, and histopathology of rats (Jiang et al. 2017). The current findings not only reveal the great security of DCL‐TP but also consequently lay solid foundations for further development of DCL‐TP in natural antilipid oxidants.

The Ames test is rapid and sensitive, which can effectively detect the mutagenic or carcinogenic potential of the test substance in an in vitro environment (Zhang et al. 2021). In the case of different concentrations of DCL‐TP with or without S9, the number of reverse colonies in each dose group of TA97a, TA98, and TA1535 strains was not significantly different from that in the SC group (*p* > 0.05), and there was no dose–response relationship (Wu et al. 2021). The number of reverse colonies of TA100 and WP2uvrA (pKM101) strains in the high‐dose group (5000 μg/plate) was significantly lower than that in the SC group (*p* < 0.05). However, this phenomenon was considered that DCL‐TP has antibacterial or antimutagenic effects in high dose. It has been reported that the antibacterial activity of the ethanol extract of *C. longepaniculatum* is mainly concentrated in the medium polarity ethyl acetate, n‐butanol fraction, and the volatile oil with small polarity, and there is no obvious antibacterial activity in the water extract phase with large polarity (Zhang et al. 2011). On the other hand, peroxide free radicals (Pryor 1986) and hereditary toxic substances (Ba and Vasseur 1999) are potential mutagens or carcinogens. A large number of studies have shown that phenolic compounds have antioxidation, antitumor and anticancer effects. Therefore, it is preliminarily inferred that DCL‐TP with higher polarity may have antimutagenicity, and its antimutagenicity needs further experimental verification. After two Ames tests, DCL‐TP did not perform mutagenicity.

Crea as an important blood biochemical index reflecting whether the glomerular filtration rate is normal or not, is often used to evaluate kidney function (Kim et al. 2006). In this experiment, although the Crea index of the male high‐dose group was significantly different from that of the NC group (*p* < 0.05), and the average difference was only 4.3 μmol/L. The Crea index value of the male high‐dose group was still within the range of 95% reference value (Kong, Liu, and Wang 2021). This result may be due to the final operation of the male high‐dose group during the blood collection process. During the waiting period, no drinking water was given, and the blood concentration and viscosity increased, so the Crea index value in the blood was slightly higher; at the same time, there was no damage or lesion in the renal tubules and glomeruli of male rats in the pathological section photograph Therefore, it is not considered that the change in the Crea index value has toxicological significance.

The results of this study will not only improve our understanding of the activity of the DCL‐TP but also deepen our understanding of the safety of DCL‐TP. The present results provide a promising approach for the applications of DCL‐TP in the field of antilipid oxidants. Importantly, this study also lays a foundation for further development of DCL‐TP in daily healthcare applications.

## 5 Conclusion

In conclusion, this study evaluated the acute toxicity study in mice, three genetic toxicity tests, and 28‐day oral toxicity study of DCL‐TP in KM mice and SD rats. In the acute toxicity study, a single oral dose of 10 g/kg bwt DCL‐TP caused no adverse effects. DCL‐TP has no mutagenicity in the Ames test, has no mutagenic effect on the micronucleus rate of mouse bone marrow cells, and does not cause chromosome aberration of spermatocytes in mice in the dose range of 5 g/kg bwt. In the 28‐day oral toxicity study, no obvious clinical symptoms or evidence of organ‐specific toxicity in SD rats at a daily dose up to 5 g/kg bwt. The results indicated that the oral consumption of DCL‐TP was very safe for KM mice and SD rats, with a no observed adverse effect. Further chronic toxicological evaluation (Njinga et al. 2020) involved in more physiological parameters would be needed to determine its safety and application value.

## Author Contributions

**Shuting Li:** conceptualization (equal), methodology (equal), supervision (equal), writing – original draft (equal), writing – review and editing (equal). **Xi Zhou:** data curation (equal), software (equal). **Yu Jiang:** investigation (equal), supervision (equal). **Manling Fu:** investigation (equal). **Yefei Yuan:** project administration (equal), supervision (equal), validation (equal), writing – review and editing (equal).

## Conflicts of Interest

The authors declare no conflicts of interest.

## Data Availability

Data will be made available on request.

## References

[fsn370008-bib-0001] Nguyen‐Ba, G. , and P. Vasseur . 1999. “Epigenetic Events During the Process of Cell Transformation Induced by Carcinogens.” Oncology Reports 6, no. 4: 925–932. 10.3892/or.6.4.925. 10373683

[fsn370008-bib-0002] Chao, M. , R. Jia , N. Jiang , C. Tao , Q. Wei , and Z. Ying . 2013. “Analgesic Effects of Essential Oils Purified From *Cinnamomum Longepaniculatum* .” Guihaia 33, no. 4: 552–555.

[fsn370008-bib-0003] Chen, X. , L. Bi , S. Li , X. Chen , Z. Zhao , and K. Mo . 2021. “Oil Cell Morphology, Essential Oil Constituents and Antioxidant Activity of *Cinnamomum Longepaniculatum* Leaves Harvested in Spring and Autumn.” Chemistry and Industry of Forest Products 41, no. 1: 38–44.

[fsn370008-bib-0004] Du, Y. , G. Ao , Q. Wei , X. Li , and X. Niao . 2014. “Preliminary Chemical Analysis and Total Flavonoids Assaying of De‐Oiled Leaves From *Cinnamomum Longepaniculatum* .” Heilongjiang Agricultural Sciences 10: 120–123.

[fsn370008-bib-0005] Du, Y. , G. Ao , Q. Wei , X. Liao , and X. Li . 2016. “Extraction Technology and Anti‐Radical Activities In Vitro of Total Flavonoids From *Cinnamomum Longepaniculatum* Leaves.” Food Research and Development 37, no. 20: 32–36.

[fsn370008-bib-0006] Du, Y. , G. Du , Q. Wei , Y. Zeng , and Y. Li . 2015. “Extraction and Anti‐Free Radical Activity of Polysaccharides From Cinnamomum Longepaniculatum Leaves.” Food and Fermentation Industries 41, no. 5: 209–213. 10.13995/j.cnki.11-1802/ts.201505038.

[fsn370008-bib-0007] Feng, Y. , J. Bi , M. Luo , J. Wang , and Y. Chen . 2023. “Study on Antibacterial and Antioxidant Effects of Camphor Leaf Essential Oil.” Journal of Sichuan University of Science & Engineering 36, no. 3: 36–41. https://kns.cnki.net/kcms2/article/abstract?v=aKxFI3wG76gbvuY‐q89gnFGlArLhi1B_OJfukHMSgQ7t603nDUSpwpcN99jfy5OF0m41syhSLueP8WO10zapsJoKIN8oV74c7rBx_kw0puXPoy4bc_UtOR5ecWfxcFku‐9ZWpRIUqc6dWzTJxCTnXxJvGh6aNy6Z4DMRdw5A2wOx9VU9VS9xcdsK3AsC_p8AzGkKOgV3Bpo=&uniplatform=NZKPT&language=CHS.

[fsn370008-bib-0008] Wang, R. , S. Ding , D. Zhao , Z. Wang , J. Wu , and X. Hu . 2016. “Effect of Dehydration Methods on Antioxidant Activities, Phenolic Contents, Cyclic Nucleotides, and Volatiles of Jujube Fruits.” Food Science and Biotechnology 25, no. 1: 137–143. 30263249 10.1007/s10068-016-0021-y PMC6049381

[fsn370008-bib-0009] 2014. “National Food Safety Standard Chromosomal Aberration Test of Spermatogonia or Spermatocytes in Mice.” https://kns.cnki.net/kcms2/article/abstract?v=aKxFI3wG76gEmIpsj1BIlfjLNFcYmqZ5U4FChEKEHlYQ6Wj2VAJijisXOR6sX1zsND1D20Ve7eMouOa_qrb0K19xNNCY4n9CSZeo7ZulEY2sTgLipy‐sTGxpQtSWc1AvEF8SNXahquwHUoEOHSmWZhncBBuOzbHLVvcNsWbmpez7YyG2We0tgcn1O4Ywi‐kj&uniplatform=NZKPT&language=CHS.

[fsn370008-bib-0010] 2014. “National Food Safety Standard Mouse Spermatocyte Chromosome Aberration Test, 12.” https://kns.cnki.net/kcms2/article/abstract?v=aKxFI3wG76gPJNTI_lTg5pgjogdcjZakHnlJcVmeYiDq9vZpe3UNZR_bV76wudlDNBTIhExug52hXskP1BiBOFdWbeLv2lUsU8hsOFk6ZN‐JH36lGqhGgJ2Id‐JTUHYmY3K‐4esseB_jmFuZ5sZlYae1GedTIYBgh2hxg850E_BOV5zEAkE0EhbPBLc7HEIl&uniplatform=NZKPT&language=CHS.

[fsn370008-bib-0011] 2014. “National Food Safety Standard Bacterial Reversion Mutation Test.” https://kns.cnki.net/kcms2/article/abstract?v=aKxFI3wG76gPJNTI_lTg5pgjogdcjZakHnlJcVmeYiDq9vZpe3UNZSXy9PTKZxQatlwommBMNAnuQ_vnpAYNvSnBwPqBLS6WRWxycLA8y1NsgAT1FJoOvqkFzsRXRnDmHr3kEqMev7x1sPUw‐SZtd9f4vL0t9w‐p8ME8GyEC6vmoTY‐5JLJlCL6CvY3ov5mg&uniplatform=NZKPT&language=CHS.

[fsn370008-bib-0012] 2014. “National Food Safety Standard Food Safety Toxicology Evaluation Procedure, 12.” https://kns.cnki.net/kcms2/article/abstract?v=aKxFI3wG76gwt‐3OtYCc6iOLVPO58Sbpt_VM2Z9rzgbCE7bWq5OOB7x96yFq1MRXrKs7ax_PaQjmomXtVC_cqKiO8QAbkoM3EcNkHyPU84UJwhXHx9ysdkF5DptWBMI_Y9JAOB5PqzF9jtpHUKymHBtZSQ0IntppM1bphWGTySrVkoRAyYQDRNn‐HpyKffT1&uniplatform=NZKPT&language=CHS.

[fsn370008-bib-0013] 2014. “National Food Safety Standard Acute Oral Toxicity Test, 28.” https://kns.cnki.net/kcms2/article/abstract?v=aKxFI3wG76jdvn7vqaYNRibL3aeJq0GEN_lOD9g40‐EbYUHIj99L7_zJxm89XPECw2__XUHSxNVTvDX0dv3OUaAAe33uRS4NFWqkSp3LLOZL7XoA‐81MPni1m7zGG9C68gnUvBMcJ1d6LyOj4gVY1IbndXGrXZzLRpkT4Dq‐JxGv8d2B5JG8soSvTT4CztIl&uniplatform=NZKPT&language=CHS.

[fsn370008-bib-0014] Hu, W. , H. Luo , and C. Dai . 2019. “Advance in Chemical Compositions of *Cinnamomum Longepaniculatum* Leaves and Their Biological Activities.” Journal of the Chinese Cereals and Oils Association 34, no. 11: 140–146. https://link.cnki.net/urlid/11.2864.ts.20191010.1700.056.

[fsn370008-bib-0015] Hu, W. , L. Zhu , S. Ying , and S. Zhou . 2019. “Chemical Constituents and Antioxidant Capacity of the Essential Oil and Its Distillations From *cinnamomum Longepaniculatum* Leaves.” Journal of Jinggangshan University 40, no. 5: 26–33. https://kns.cnki.net/kcms2/article/abstract?v=aKxFI3wG76hmmkshcYCLzW0m_23vKl4uxQaMx4aQ4ANDZE‐WJH4YQRM2iTtTrf7ElIPyG_kac4gEU9‐Cc31hy0LzLDLiacjL7_kDymzLcDE8c‐qYTnvT7sHrm1F4qIFp9vC92Iy4c0V5gXboyNEPsZK4VtKAed2XAwgmgovMIcoUlm2DkXV19aZp6WTDKBW2x_cL‐J33v0I=&uniplatform=NZKPT&language=CHS.

[fsn370008-bib-0016] Jiang, P. , Z. Ni , B. Wang , et al. 2017. “Acute Toxicity, Twenty‐Eight Days Repeated Dose Toxicity and Genotoxicity of Vanadyl Trehalose in Kunming Mice.” Regulatory Toxicology and Pharmacology 85: 86–97. 10.1016/j.yrtph.2017.02.001. 28202346

[fsn370008-bib-0017] Kahl, R. , and H. Kappus . 1993. “Toxicology of the Synthetic Antioxidants BHA and BHT in Comparison With the Natural Antioxidant Vitamin E.” Zeitschrift für Lebensmittel‐Untersuchung und ‐Forschung 196, no. 4: 329–338. 10.1007/bf01197931. 8493816

[fsn370008-bib-0018] Kim, S. B. , D. W. Lee , C. I. Cheigh , et al. 2006. “Purification and Characterization of a Fibrinolytic Subtilisin‐Like Protease of *Bacillus subtilis* TP‐6 From an Indonesian Fermented Soybean, Tempeh.” Journal of Industrial Microbiology & Biotechnology 33, no. 6: 436–444. 10.1007/s10295-006-0085-4. 16470353

[fsn370008-bib-0019] Kong, Q. , H. Liu , and J. Wang . 2021. “Discussion on the Background Reference Value Range of Hematology, Serum Biochemistry and Hemagglutination Index of SD Rats and Beagles.” Drug Evaluation Research 44, no. 12: 2601–2607.

[fsn370008-bib-0020] Lin, X. 2020. “In the Core Literacy Perspective,the Development and Practice of School‐Based Course Named Camphor Tree of Hometown. Sichuan Normal University.” 10.27347/d.cnki.gssdu.2020.000870.

[fsn370008-bib-0021] Njinga, N. S. , A. T. Kola‐Mustapha , A. L. Quadri , et al. 2020. “Toxicity Assessment of Sub‐Acute and Sub‐Chronic Oral Administration and Diuretic Potential of Aqueous Extract of *Hibiscus sabdariffa* Calyces.” Heliyon 6, no. 9: e04853. 10.1016/j.heliyon.2020.e04853. 33005778 PMC7511736

[fsn370008-bib-0022] Pryor, W. A. 1986. “Cancer and Free Radicals.” Basic Life Sciences 39: 45–59. 10.1007/978-1-4684-5182-5_4. 3767848

[fsn370008-bib-0023] Ren, C. , X. Wang , X. Wang , and Y. Gahafu . 2021. “Study on the Techniques of Purification for the Phenolic Acid Constituents From Roots of *Salvia Deserta* Schan. *Northwest* .” Pharmaceutical Journal 36, no. 4: 527–532. https://kns.cnki.net/kcms2/article/abstract?v=Z‐eERPAUDzzv9raKNBQS_TF9MdHPSPLnkNeZhX6rmmLC5peJy5XFpoVydr35890D2daQ5aMmv4wC5OhOKcurNOcOYad6iFvgMjZbWMqf8PZ3vROEsYpF2opIBJ3pAmS077Z80O9ZERFevx4xPUXXlV0tz1nt3Pw6RF2YPAs1EtfKmvMlkRMvYnSqHrv_X4L4BL5Y8amaKTg=&uniplatform=NZKPT&language=CHS.

[fsn370008-bib-0024] Tao, C. 2011. “Study on Antibacterial, Antifungal,Analgesic and Anti‐Inflammatory Activity and Anti‐Inflammatory Mechanism of the of Extraction of *Cinnamomum Longepaniculatum*(Gamble) N.Chao. Sichuan Agricultural University.”

[fsn370008-bib-0025] Wu, J. , Y. Zhang , Z. Lv , P. Yu , and W. Shi . 2021. “Safety Evaluation of *Aloe vera* Soft Capsule in Acute, Subacute Toxicity and Genotoxicity Study.” PLoS One 16, no. 3: e0249356. 10.1371/journal.pone.0249356. 33770149 PMC7997006

[fsn370008-bib-0026] Xiao, S. , D. Hu , Y. Gao , et al. 2021. “Safety Assessment of Subtilisin QK in Rats.” BMC Pharmacology and Toxicology 22, no. 1: 38. 10.1186/s40360-021-00506-w. 34172094 PMC8235616

[fsn370008-bib-0027] Ying, H. , C. Wang , J. Yue , S. Jiao , X. Wang , and L. Jiang . 2020. “Extraction of Cinnamomum Longipaniculatum Essential Oil and Its Chemical Composition, Antioxidation and Antibacterial Properties.” Storage and Process 20, no. 4: 183–191.

[fsn370008-bib-0028] Yuan, K. 2021. “Research on Separation and Purification of Main Components From Cinnamomum Longepaniculatum Oil. Sichuan University.” 10.27342/d.cnki.gscdu.2021.006805.

[fsn370008-bib-0029] Zhang, C. , Q. Wei , Y. Du , L. Zhou , Q. Jiang , and Z. Yin . 2011. “The Antibacterial Study on Extracts From De‐Oiledleaves of *Cinnamomum Longepaniculatum* Against Three of the Pathogenic Bacteria.” Guihaia 31, no. 5: 690–694. https://kns.cnki.net/kcms2/article/abstract?v=aKxFI3wG76heFc56VLp51b4yOh8Q9gjMzBbtOhDERBCKfU‐‐17cjihoLUBRFowFe4cNp2nSO3phHBmKWtHFl7HT2sFhDuVeArprsAZKaYEfREY58__eQ32EHUZg2iQv0GlEwvgQsNPFJPyC_CfaoW06qhW_To0CAKYiZi9QtTYrlIZK6aYuG55409xp_MHsY&uniplatform=NZKPT&language=CHS.

[fsn370008-bib-0030] Zhang, Q. , M. Wang , Y. Ma , Y. Sun , and S. Wang . 2021. “Cytoprotective Effect and Safety of Morchella Esculenta Protein Hydrolysate and Its Selenized Derivative.” Food and Fermentation Industries 47, no. 10: 116–123. 10.13995/j.cnki.11-1802/ts.025831.

[fsn370008-bib-0031] Zhang, Y. , F. Shi , and Y. Yang . 2017. “Determination of the Total Polyphenols Content in Grape Spirits by Folin‐Ciocalteu Colorimetry Method.” China Brewing 36, no. 10: 163–166. https://link.cnki.net/urlid/11.1818.TS.20171026.1605.070.

[fsn370008-bib-0032] Zhang, Y. , Y. Xi , H. Ge , X. Feng , and H. Ma . 2016. “Determination of Total Polyphenols in Calyx Physalis by Folin‐Ciocalteu Method.” Food Research and Development 37, no. 23: 138–141. https://kns.cnki.net/kcms2/article/abstract?v=Z‐eERPAUDzwokEIKqgw1iYubTPp7Ydg8wwd8Bnbnn8F4pxuObwb15Lvi0qPs4GXl77b_Htlk2NcPnCtDIF8P8LWwspWBrf78fYVayEGw_dpyVjEyheSNwS26c5JKg3KIG6Z6h‐8q4yYEYZ6P1B1gvOPjPdlJENvZZj5gWJsUlALxFLhjbYL4q8rKC1mZsQIO‐79uVdAOBjs=&uniplatform=NZKPT&language=CHS.

[fsn370008-bib-0033] Zhou, C. , and D. Lian . 2020. “Yibin Oil Camphor Industry Development Status and Countermeasures.” Agriculture and Technology 40, no. 16: 65–68. 10.19754/j.nyyjs.20200830023.

[fsn370008-bib-0034] Zhou, W. , W. Zhou , J. Bei , Y. Wang , S. Ruan , and L. Nin . 2022. “Separation and Identification of Aqueous Extracts of Cinnamomum Longe‐Paniculatum Leaves and Their Anti Hepatocellular‐Cancer Activity.” Chemistry and Industry of Forest Products 42, no. 6: 1–10.

[fsn370008-bib-0035] Zhou, X. , Y. Jiang , and Y. Yuan . 2024. “Optimization of Extraction and Purification Process of Total Phenols From Deoiled Cinnamomum Longepaniculatum Leaves and Its Antioxidant Study In Vitro.” Chinese Journal of Modern Applied Pharmacy 41, no. 11: 1499–1507. 10.13748/j.cnki.issn1007-7693.20231001.

[fsn370008-bib-0036] 2014. “National Food Safety Standard 28‐Day Oral Toxicity Test.” https://kns.cnki.net/kcms2/article/abstract?v=aKxFI3wG76gEmIpsj1BIlfjLNFcYmqZ5U4FChEKEHlYQ6Wj2VAJijpyrtdxKyCgquf8r67hRNpuaCwgm4JZjKRTwps7V1mEHTRsWrSBs4LljXNyJBTJj9CGtWlJOIq0RWznYMdaTlK8JLun4v3XaWMM92DewZ5IZe_hJ8PKQwAwyXI4lyu_5WpAQNBVQpRIe&uniplatform=NZKPT&language=CHS.

